# Computerized versus hand-scored health literacy tools: a comparison of Simple Measure of Gobbledygook (SMOG) and Flesch-Kincaid in printed patient education materials

**DOI:** 10.5195/jmla.2018.262

**Published:** 2018-01-02

**Authors:** Kelsey Leonard Grabeel, Jennifer Russomanno, Sandy Oelschlegel, Emily Tester, Robert Eric Heidel

## Abstract

**Objective:**

The research compared and contrasted hand-scoring and computerized methods of evaluating the grade level of patient education materials that are distributed at an academic medical center in east Tennessee and sought to determine if these materials adhered to the American Medical Association’s (AMA’s) recommended reading level of sixth grade.

**Methods:**

Librarians at an academic medical center located in the heart of Appalachian Tennessee initiated the assessment of 150 of the most used printed patient education materials. Based on the Flesch-Kincaid (F-K) scoring rubric, 2 of the 150 documents were excluded from statistical comparisons due to the absence of text (images only). Researchers assessed the remaining 148 documents using the hand-scored Simple Measure of Gobbledygook (SMOG) method and the computerized F-K grade level method. For SMOG, 3 independent reviewers hand-scored each of the 150 documents. For F-K, documents were analyzed using Microsoft Word. Reading grade levels scores were entered into a database for statistical analysis. Inter-rater reliability was calculated using intra-class correlation coefficients (ICC). Paired *t*-tests were used to compare readability means.

**Results:**

Acceptable inter-rater reliability was found for SMOG (ICC=0.95). For the 148 documents assessed, SMOG produced a significantly higher mean reading grade level (M=9.6, SD=1.3) than F-K (M=6.5, SD=1.3; *p*<0.001). Additionally, when using the SMOG method of assessment, 147 of the 148 documents (99.3%) scored above the AMA’s recommended reading level of sixth grade.

**Conclusions:**

Computerized health literacy assessment tools, used by many national patient education material providers, might not be representative of the actual reading grade levels of patient education materials. This is problematic in regions like Appalachia because materials may not be comprehensible to the area’s low-literacy patients. Medical librarians have the potential to advance their role in patient education to better serve their patient populations.

## INTRODUCTION

Medical librarians have played a crucial role in patient education for nearly a century. References to terms such as bibliotherapy, social hygiene, and consumer health are easily found in the literature to give a historical perspective [1–3]. However, the role of the medical librarian today has expanded to become embedded in both the academic and medical setting. In some settings, librarians are a part of the patient safety and quality improvement teams, and in provision of patient-and-family centered information to patients [4, 5]. In many settings, nurses depend on medical librarians to teach them the art of evaluating health information for patient care [6]. Librarians also impact patient care by teaching health care providers how to evaluate patient education material and by providing services to determine if the reading grade level of material is appropriate for the patient population.

Printed patient education materials are important aspects in patients’ recovery in that they reinforce verbal communication and vital care instructions that patients receive from health care providers [7]. Goal two of the US Office of Disease Prevention and Health Promotion’s “National Action Plan to Improve Health Literacy” promotes changes in health care systems that “improve health information, communication, and informed decision-making” [8]. Distributing patient education materials that are comprehensible to all patients is essential to achieving this national goal.

Research has indicated that patient education materials influence patient compliance. Mundt and colleagues studied the impact of patient education on patients’ adherence to instructions and medication regimes. They found that the compliance rate for patients who completed the follow-up assessments were higher than for those who received normal care [9]. Another study concluded that patients who received educational materials on their aromatase inhibitor treatment for a specific form of breast cancer had a higher rate of compliance to physician recommendations for treatment than those who did not receive any educational material [10]. Lastly, a longitudinal study on asthma medication adherence, in which patients received an audiotape and educational booklet, determined that this intervention had a beneficial effect on asthma medication adherence over time [11].

Given the impact of patient education materials on patient compliance, the grade level at which the materials are written is crucial. The average American reads at the eighth- to ninth-grade level, while one of five reads at the fifth-grade level [12]. The National Institute of Health (NIH) and the American Medical Association (AMA) both recommend patient education materials be written at or below a sixth-grade reading level [13, 14], while the Joint Commission [15] recommends materials be written at a fifth-grade level or lower. Despite these recommendations, patient education materials continue to be written at grade levels beyond the average American’s literacy skills [16].

Numerous readability formulas are available when assessing reading grade level of patient education materials. Simple Measure of Gobbledygook (SMOG) is an assessment tool that utilizes a hand-scored method. SMOG allows an evaluator to determine the grade level of patient education by counting 10 sentences at the beginning, the middle, and the end of a document. The evaluator counts every word of 3 or more syllables in those 30 sentences [17]. The number of syllables in each section is then totaled and converted to a corresponding reading grade level score [18]. Previous research suggests that SMOG is a useful tool when completing the reading grade level and predicts 100% comprehension [19].

The computerized Flesch-Kincaid (F-K) grade level method is another commonly used reading grade level assessment tool. F-K grade level is added to Microsoft Word through the Spelling and Grammar tool, in which readability statistics are displayed. Once the document is put through Microsoft’s spell check feature, the readability statistics are automatically calculated and displayed. Patient education material providers often use this tool because of its ease of use and computerized platform. However, F-K grade level tends to predict lower reading grade level scores [20], and research has consistently found this to be true. Walsh and Volsko evaluated the reading grade level of Internet-based patient education on the top 5 causes of death in the United States and found that F-K grade level scored 2 to 3 grade levels lower than SMOG [21]. Additionally, Freda found that F-K grade level consistently scored patient education brochures 2 to 3 grade levels lower than SMOG [22].

Hand-scoring tends to be more reliable than computer scoring, since computerized scoring can be misleading based on the text provided in each document [23]. Computer programs use different methods to count sentences, words, and syllables, which can cause discrepancies in grade level [20]. By hand-scoring, evaluators are working directly with the text, which alerts evaluators to longer sentences and multisyllabic words as well as sentence structure and ease of reading [23]. Wang and colleagues found that the F-K grade level was the most commonly used readability formula, but SMOG was the most consistent in performance and the most practical in the health care setting [24].

**Figure 1:**
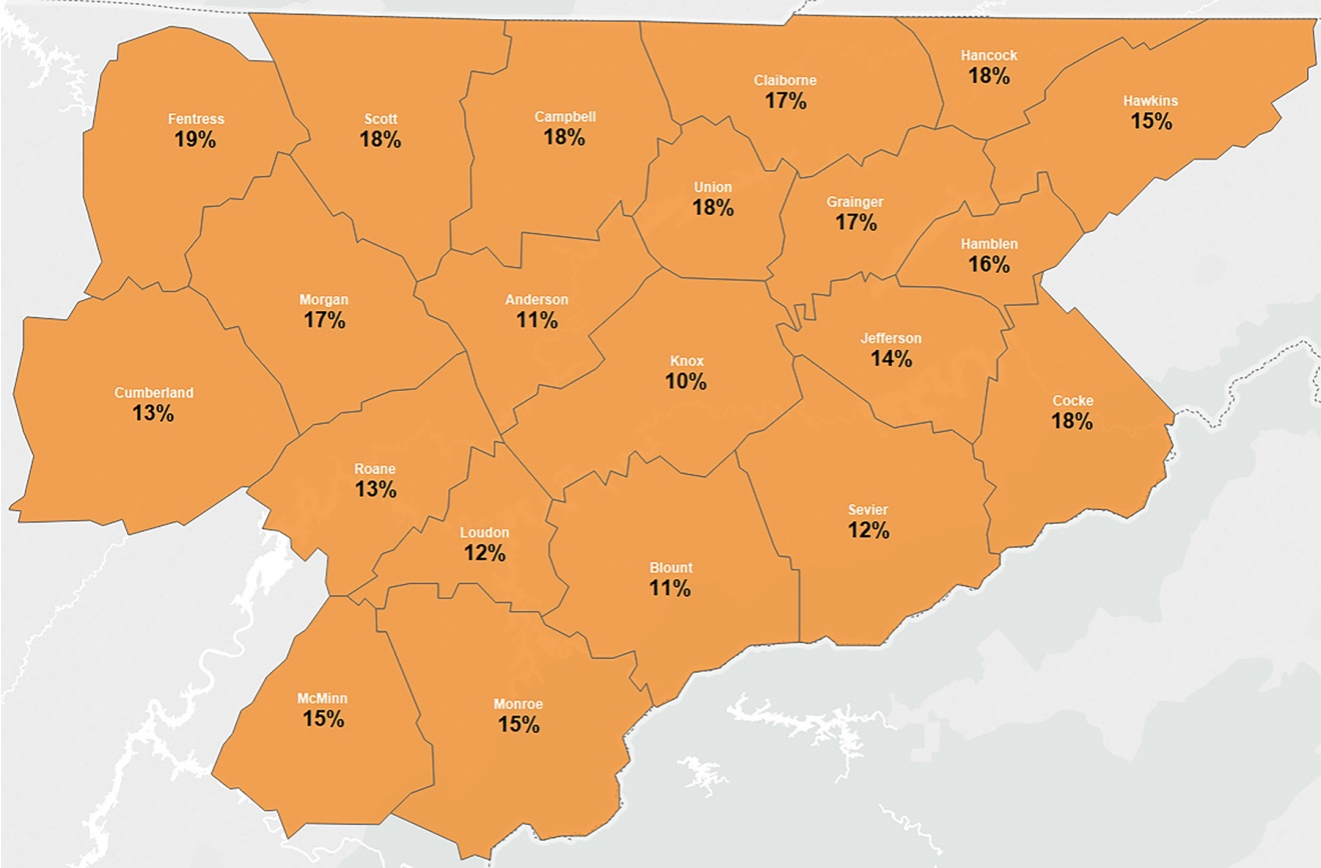
Counties served by University of Tennessee Medical Center and percentage of the population who lack basic prose literacy skills

Appropriate reading grade levels for patient education materials are of extreme importance in low literacy areas of the United States, including Appalachia. The Appalachian region has historically lagged behind average US literacy levels, with nearly 30% of adults living in the region considered “functionally illiterate” [25]. In addition, the educational attainment in the Appalachia region is lower than the national level, and fewer students obtain a bachelor’s degree [26]. The study location for this project, the University of Tennessee Medical Center (UTMC), is located in the heart of Appalachia, Tennessee. In the 21-country region serviced by UTMC (Figure 1), 10%–19% of the population lacks basic prose literacy skills [27]. Additionally, UTMC, like many US hospital systems, outsources its patient education materials to a third party provider, ExitCare, a patient education material provider owned by Elsevier. ExitCare markets their patient education materials as written “at a fifth-to eighth-grade reading level, with easy-to-read education written at a fourth-grade reading level or below” [28].

The purpose of this study was to compare and contrast reading grade level scores of UTMC’s patient education materials using hand-scored and computerized methods and to determine if these materials adhered to both the vendor’s published reading grade levels and the nationally recommended sixth-grade reading level. Although studies have addressed F-K grade level versus SMOG in evaluating patient education, this study reviewed the importance of using a reliable readability tool that would accurately portray the reading grade level and highlighted the differences in using a computerized method versus a hand-scoring method when evaluating patient education. Additionally, this study focused on the Appalachian population and how librarians could play a role in advocating for quality patient education.

## METHODS

Both SMOG and F-K grade level assessment methods were used in this study. To assess the readability of patient education materials distributed at UTMC, the authors received a list from the Patient Education Committee of the 150 most distributed patient education documents from January 2016 through May 2016. These documents, which covered a broad base of health topics, were then downloaded from UTMC’s internal system. Each document was assigned a unique identification number to be used for tracking and data entry. These materials included 137 documents created by the vendor and 13 custom documents created internally by UTMC staff. Twenty-two of the 137 vendor documents were labeled “easy-to-read.” However, F-K grade level could not be obtained on 2 of the documents because they were images; therefore, the sample size for SMOG versus F-K grade level was n=148.

Graduate students from the University of Tennessee–Knoxville, who were trained in SMOG assessment, reviewed the patient education materials. Each document was analyzed by three independent reviewers. Results from each reviewer were collected, reviewed, and tallied by the principle investigator (PI), a librarian. F-K grade level scores were determined by the PI, who entered each document into the existing F-K tool available in Microsoft Word. Data were checked for coding and data entry errors by the librarian to ensure data validity. Data from both the SMOG and F-K analyses were entered into SPSS version 21 (Armonk, NY: IBM) for further analysis.

The SMOG score for each document was calculated by averaging the 3 independent reviewers’ SMOG scores. The 3 independent SMOG ratings were analyzed for inter-rater reliability using 1-way random intra-class correlation coefficients (ICCs). These continuous variables from the SMOG analysis were tested for normality using skewness and kurtosis statistics. Any skewness or kurtosis statistic above an absolute value of 2.0 assumed a non-normal distribution. The distribution of differences between the SMOG and F-K grade level scores were also checked for normality and outliers. A paired *t*-test was used to compare the SMOG scores and F-K grade level scores for all documents. Statistical significance was assumed at an alpha value of 0.05.

## RESULTS

We found excellent inter-rater reliability between SMOG reviewers (ICC=0.95). The average SMOG scores were normally distributed. For the 148 assessed documents, SMOG produced a significantly higher mean grade reading level (mean [M]=9.6, SD=1.3) than F-K grade level (M=6.5, standard deviation [SD]=1.3) (*t*(147)=56.56, *p*<0.001, 95% confidence interval [CI] of difference 2.96–3.17). SMOG test results showed that reading grade levels were higher than the national recommended reading grade level average for both custom and vendor documents.

Using SMOG, 13 of 13 (100.0%) of the custom-designed patient education materials scored above the nationally recommended sixth-grade reading level. All of the “easy-to-read” documents (n=22) created by the vendor scored above the company’s promoted maximum fourth-grade reading level. For standard vendor documents, 115 of 115 documents (100.0%) scored above the company’s promoted maximum eighth-grade reading level, and 77% (n=115) scored above the nationally recommended sixth-grade reading level.

**Figure 2:**
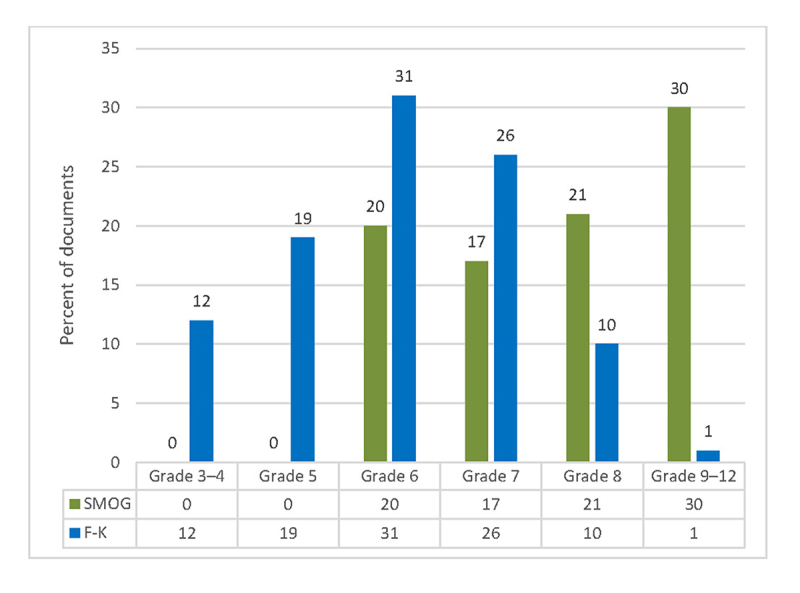
Simple Measure of Gobbledygook (SMOG) versus Flesch-Kincaid (F-K) reading grade level (n=148)

**Figure 3:**
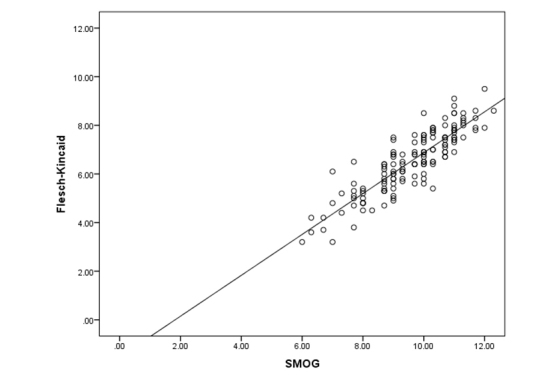
Scatterplot of SMOG*Flesch-Kincaid

When assessing the same materials using F-K grade level, 9 of 11 custom documents (81.8%) scored above the nationally recommended sixth-grade reading level. For the vendor’s “easy-to-read” documents, 17 of 22 (77.3%) scored above the company’s stated maximum fourth-grade reading level. For standard vendor documents, only 12 of 115 (10.4%) scored above the company’s stated maximum eighth-grade reading level, while 92 of 115 (80.0%) scored above the nationally recommended sixth-grade reading level (Figure 2). A scatterplot of SMOG versus F-K grade level scores is shown in Figure 3.

## DISCUSSION

The patient education documents evaluated in this study covered a wide spectrum of topics in the most commonly distributed materials at UTMC. Assessing patient education materials at UTMC produced significantly different results when using a hand-scored method, SMOG, and a computerized method, F-K grade level. When SMOG was used, 77% of materials were above the nationally recommended sixth-grade reading level, while 58% scored above eighth grade. When using the computerized F-K grade level, documents produced a significantly lower grade level score as opposed to SMOG.

This result is consistent with D’Alessandro and colleagues, who concluded that patient education materials distributed on the Internet produced significantly higher reading grade level scores when they used SMOG assessments as opposed to F-K grade level [29]. The findings are also consistent with Walsh and Volsko [21] and Freda [22], who found SMOG to be 2 to 3 grade levels higher than F-K grade level. Additionally, the Centers for Medicare & Medicaid Services, US Department of Health and Human Services, warns that “Flesch-Kincaid scores tend to underestimate actual reading grade level because they are often several grade levels below results obtained using other measurements” [30]. One potential explanation for the discrepancy between SMOG and F-K grade level is that the formula used in F-K grade level only allows a maximum of a twelfth-grade reading level score [20]. This systematic limitation could lessen the actual reading grade level score, as values of thirteen and higher are not possible.

Hand-scoring patient education materials allows evaluators to work directly with the text, alerting them to multisyllabic words and long sentences [23]. Computerized-scoring tends to be easier to use; however, as previously mentioned, patient education materials tend to score lower in reading level than they are. Computerized-scoring is highly dependent on period placement. For example, document number 30 in this study had only 1 period at the very end of the document, causing the F-K grade level to be 6.7. However, once periods were added to the end of each sentence, the score decreased to 5.8, further proving the use of periods are of the utmost importance when using the computerized method. Therefore, researchers found the hand-scoring method SMOG to be the better method to assess patient education at UTMC.

A lack of comprehension of print patient education materials can negatively impact a patient’s health literacy [31]. Patients with low health literacy have increased readmission rates, frequently return to the emergency department for the same conditions, and are less able to manage their chronic conditions [32–34]. To help increase patient understanding and help combat these issues, the quality of written communication needs to be improved. Research has shown that “using materials that are written in a manner that facilitates the uptake and use of patient education content has great potential to improve the ability of patients and families to be partners in care and to improve outcomes, especially for those patients and families with limited general literacy or health literacy skills” [35].

Therefore, print communication needs to be written at a reading grade level that is understandable to people of all literacy levels. With this in mind, librarians can advance their role and help to ensure that patient education materials are written at a grade level that is understandable to all. As teachers, educators, and researchers in a health care setting, it is important that librarians understand the different reading grade level tools and be able to teach nurses and health care providers how to accurately find the reading grade level of patient education materials.

Medical librarians’ role is constantly changing in that they are not only providing support for staff, but also providing support for patients who need education [36]. Librarians can take a more active role in evaluating patient education distributed at their medical centers to determine if it is at the required reading grade level. This can be done through participating on a hospital’s patient education committee or teaching classes on evaluating grade level to nurses, hospital staff, medical students, and residents. At the UTMC, a hospital librarian evaluates in-house created materials for grade level due to involvement in the Patient Education Committee [37]. Through working with the Patient Education Committee, the librarian can advocate for the patient’s right to have patient education materials written at a lower reading grade level.

Medical librarians have the potential to initiate research on lowering the reading grade level of patient education materials, such as this study has done. Research can include using focus groups of lay people to review patient education materials for understandability and readability. For example, Blanck and Marshall discussed how a librarian was able to get their Patient Education Committee to test patient education materials with a panel of lay people [38]. Future work is being conducted by librarians who worked on this study to edit the patient education materials that they reviewed and, as a result, lower the reading grade level.

There were several limitations to this study that should be noted. First, by design, the most utilized patient education materials were selected for analysis in a nonrandomized fashion. This limited our ability to infer “causal effect” due to lack of document randomization. It cannot be said with certainly that the results of this nonrandomized sample would be duplicated if all documents distributed by UTMC were analyzed. Future research should consider using a randomized sample of patient education materials from various geographical regions and institutions around the United States.

The patient education materials in this study that are being distributed at UTMC do not meet the current NIH, AMA, or Joint Commission recommendations for reading grade levels in the United States. These results will interest libraries whose institutions have chosen this vendor to provide patient education materials. Results produced by computerized methods produced significantly lower reading grade levels than hand-scored methods. While computerized methods may be more cost-effective and efficient than hand-scored methods, third-party patient educational material providers and health care providers should be cautioned that the scores produced by this method may be manufacturing reading grade levels that seem lower than they actually are.

Health care providers in low literacy areas, like Appalachia, should be particularly concerned about reading grade levels of materials distributed, given the region’s high percentage of individuals with low literacy. Medical librarians have the potential to make an impact on lowering the reading grade level of patient education materials through teaching, being on a patient education committee, and conducting future research.
